# Health Beliefs and Perceptions of Trachoma in Communities on the Bijagos Archipelago of Guinea Bissau

**DOI:** 10.3109/09286586.2015.1036889

**Published:** 2015-07-09

**Authors:** Katie Thompson, Harry Hutchins, Aramata Baio, Eunice Cassama, Meno Nabicassa, Robin Bailey, Anna R. Last

**Affiliations:** ^a^ University of Birmingham Medical School, Birmingham, UK; ^b^ Estudo do Tracoma, Guinea Bissau (c/o London School of Hygiene & Tropical Medicine, London, UK); ^c^ Programa Nacional de Saude de Visao, Guinea Bissau; ^d^ Clinical Research Department, London School of Hygiene & Tropical Medicine, London, UK

**Keywords:** Bijagos, Guinea-Bissau, health beliefs, hygiene, interviews, qualitative, trachoma

## Abstract

*Purpose*: The World Health Organization aims to eliminate blinding trachoma by 2020 using the SAFE strategy: Surgery for trichiasis, Antibiotics, Facial cleanliness and Environmental improvement. Trachoma is hyperendemic on the remote Bijagos Archipelago of Guinea-Bissau, West Africa. Sociocultural factors remain unexplored here, despite their potential impact on disease control, particularly through the “F” and “E” aspects. By examining these, we aim to illuminate this population's unreported health beliefs, hygiene behaviors and disease perceptions. This understanding will help to optimize future public health interventions, and guide the distribution of limited healthcare resources.

*Methods*: Two unmatched interview series were conducted 1 year apart on Bubaque Island in the Bijagos Archipelago; one in rural villages using purposive snowball sampling, the other in a semi-urban settlement, using random-cluster sampling. Interviews were conducted and recorded in Kriolu, the local dialect, by a supervised local field assistant before translation into English for conventional content analysis.

*Results*: Trachoma was unheard of in either series, despite ongoing local trachoma research. A heterogeneous range of disease etiology and preventative measures were suggested, but the importance of hygiene was more widely reported by semi-urban interviewees. Although western medicine was well regarded, traditional practices continued, particularly in the rural populations.

*Conclusions*: Differences in knowledge, beliefs and behaviors were apparent between the two series. Despite widespread rudimentary knowledge of disease prevention, targeted education might benefit both communities, particularly basic hygiene education for rural communities. Healthcare access should also be improved for rural populations. The impact of these measures could be assessed by future fieldwork.

## INTRODUCTION

The Bijagos Archipelago, a remote island group off the coast of Guinea-Bissau, is hyperendemic for trachoma.^1^ This neglected tropical disease is the leading infectious cause of blindness worldwide, burdening the poorest and most underdeveloped areas.^2–4^

Active trachoma, a self-limiting keratoconjunctivitis, may progress to chronic scarring disease and ultimately blindness in later life after repeated cycles of infection in childhood.^5^ The Bijagos Archipelago is hyperendemic for trachoma. The prevalence of active trachoma in children aged 1–9 years is 22.0% (95% confidence interval, CI 18.9–25.5%)^6^ and the prevalence of trichiasis, a consequence of scarring, in those over the age of 15 years is 3.5% (95% CI 2.1–4.9%; Last AR, unpublished data).

The World Health Organization (WHO) advocates the SAFE strategy to eliminate blinding trachoma by 2020: Surgery for trichiasis, antibiotics for active infection, facial cleanliness and environmental improvements.

SAFE implementation on the Bijagos has been incomplete. The population has received several rounds of community mass drug treatment in accordance with WHO and national trachoma control policies. Additionally there have been sporadic trichiasis surgery camps organized to address the burden of surgical disease. However there has been limited formal implementation of the F and E aspects of SAFE. The potential contribution of these interventions to prevalence reduction has been demonstrated in other studies, which particularly highlighted the benefits of latrine use and facial cleanliness associated with reducing trachoma transmission.^7–9^

Successfully implementing F and E aspects requires community support and behavioral changes.^10–12^ Health behaviors, such as the maintenance of facial hygiene, are heavily influenced by sociocultural factors, and cultural understanding is therefore key to the implementation of effective disease control,^13^ especially regarding community beliefs about etiology.^14^ As there is a paucity of research examining the foundations of community health behaviors and attitudes towards eye disease, this study addresses an important gap in the literature.

The objective of this research was to explore sociocultural beliefs regarding etiology, disease recognition, health-seeking behaviors and treatment practices in these trachoma-endemic Bijagos communities. This understanding may assist in targeting appropriate measures for SAFE implementation in these communities, in particular the F and E aspects.^15^^,^ 16

## MATERIALS AND METHODS

### Study Setting and Design

The Bijagos Archipelago lies off the Atlantic coast of Guinea-Bissau in West Africa (Appendix 1). Small, mostly rural communities are spread throughout the islands. While the archipelago's remoteness has preserved the inhabitants' traditional culture, the islands themselves remain largely undeveloped, with little infrastructure and limited access to healthcare and education. On Bubaque Island, the primary transport hub to the mainland, most people live in rural communities supported by subsistence agriculture and fishing, although there is one relatively diverse and itinerant semi-urban settlement, Praça, which has the weekly mainland ferry, greater access to education, trade and currency, and is the site of the hospital. Islanders speak the local Bijogo language, although Kriolu, the lingua franca, is widely spoken. An anthropologically complex society, most childcare is provided by women, despite notable gender-equality, evidenced by matriarchal elements. While not overtly religious, spirituality is a prominent part of island life, particularly in rural communities.

Two unmatched qualitative interview series were conducted on Bubaque 1 year apart. The first series primarily aimed to establish the feasibility of qualitative research in this setting, given the significant linguistic and logistical challenges. Having proven viability, the second series aimed to gather more targeted information; expanding on key points raised by the pilot work, and focusing on aspects of the SAFE strategy to produce data potentially relevant to local implementation. Between these dates, a mass treatment intervention was undertaken.

### Participants

For the first interview series (conducted in March 2012), the population was sampled exclusively from Bubaque's rural villages using a purposive-snowball technique, aiming to establish opinion from as wide a range of people as possible. Prominent community figures were identified by consensus and invited to participate, as their opinions could feasibly influence those of the rest of the village. Equally, ordinary villagers were approached to judge the homogeneity of community beliefs.

The second series (conducted in March 2013) included only the island's semi-urban population. The primary childcare provider, identified by the household as the person responsible for childcare, was interviewed in each of 13 randomly selected households. These households (defined as groups of individuals who eat from the same cooking pot) formed part of a population of 135 households randomly selected as part of a trachoma prevalence survey in Praça. Details of this survey have been described elsewhere.^6^ All households containing children under the age of 10 years were included. If caregivers were unavailable or did not give consent, further households were randomly selected from a reserve list.

The total sample size was 25: 12 from rural communities and 13 from the semi-urban settlement. The limiting factor for interview quantity was time; these figures represent the maximum number of interviews possible during the researchers' time in the field.

Demographic information is displayed in Table 1. Four participants were male and 21 female, which was unsurprising given that the second interview series focused on childcare providers, a predominately female role in Bijogo society. Age, though often uncertain, ranged from 13–80 years. Literacy was poor in most participants, several being unable to write their name; in these cases, all information was read aloud beforehand to ensure informed consent. Among the rural participants, occupations included medicine man, teacher, nurse, chiefs or elders and mothers. In the semi-urban group, most were homemakers or else small-scale traders, selling fruit or cloth. Participation in previous mass drug administration or other public health campaigns was not taken into account when selecting participants.

**TABLE 1.** Demographic information of participants interviewed on perceptions of eye conditions and trachoma, Bijagos Archipelago.

		Rural (*n* = 12)	Semi-urban (*n* = 13)	Total (*N* = 25)
Median age, years (interquartile range)		49 (35.5–60.0)	26 (18.5–33.5)	34 (24.0–51.0)
Sex, *n* (%)	Male	4 (33.3)	0 (0)	4 (16.0)
	Female	8 (66.7)	13 (100)	21 (84.0)
Ethnicity, *n* (%)	Bijogo	12 (100)	8 (61.5)	20 (80.0)
	Mandinka	0 (0)	2 (15.4)	2 (8.0)
	Balanta	0 (0)	1 (7.7)	1 (4.0)
	Fula	0 (0)	1 (7.7)	1 (4.0)
	Manjaco	0 (0)	1 (7.7)	1 (4.0)
Literate^a^, *n* (%)		5 (41.7)	11 (84.6)	16 (64.0)

^a^“Literate” is defined here as being able to write one's own name

### Data Collection

Semi-structured interviews were conducted by a trained local field assistant in Kriolu, under supervision of the researcher. All field assistants were fluent in both Kriolu and Bijogo. Recordings were made by dictaphone then translated and transcribed into English by a local translator. Semi-structured interviews were employed to allow hypothesis-formation and exploration, focusing on, but not limited to, themes anticipated from prior literature. Questions were therefore not rigid, but included the themes of disease and symptom recognition; etiology, transmission, prevention, treatment, perceptions of western healthcare and the domestic use of water (Appendices 2 and 3).

### Data Analysis

Conventional content analysis^17^ was conducted on each interview series independently before both researchers conducted conventional content analysis on all data, allowing identification of population-specific conclusions and then comparison. This involved immersion in the data, followed by descriptive coding; highlighting transcript passages which embodied a noteworthy concept. These codes were then categorized, and their relations form the basis of this work.

The quotes presented represent the major themes which emerged from this analysis. They do not encompass every opinion given, but were selected for reflecting the prevailing views of as many participants as possible. Translations are given as directly as possible to allow consideration in future research but were amended to be in fluent English. Care was taken to avoid changing their meaning. Some terms, without literal translations, have been bracketed and explained.

### Ethics Statement

This study complied with the guidelines of the Declaration of Helsinki. Ethical approval was granted by the University of Birmingham Internal Ethics Review Committee, the London School of Hygiene and Tropical Medicine Ethics Committee, the Comité Nacional de Ética na Saudé of Guinea-Bissau and the Medical Research Council, Gambia. Informed consent was obtained in Kriolu from all participants prior to interview, following a 24-hour period where opportunity was provided to ask questions of the research team. All information was treated confidentially at all times by all team members.

## RESULTS

### Knowledge of the Term “Trachoma”

I don't know trachoma … If I see a child with eye-ache, I just take him to hospital and they treat him without telling me what disease it is. (U11)

Both rural (R) and semi-urban (U) populations were unfamiliar with the term “trachoma,” however, the term “eye-ache” was frequently used, presumably to describe all visible eye disease as no direct translation exists for trachoma specifically. The term for trichiasis, however, was distinct from this and participants did not connect the two. Two respondents (8%) recognized “trachoma” but could not give the meaning: “I have heard of it, but I've never read about it or had it explained to me in depth.” (U8)

### Awareness of Ocular Pathology

With trachoma awareness low, knowledge regarding generalized eye symptoms was probed. Apart from trauma, the most commonly described problems were:

- Conjunctivitis/suppurative eye disease (*n* = 15 respondents; 60% of total): “You see *ramela* [rheum; sticky ocular discharge] sticking around their eyes.” (R10)
- Trichiasis (*n* = 9; 36%): “Eyelashes prick the eye and you cannot see. Sometimes it irritates the eye.” (R11)
- Visual impairment (*n* = 7; 28%): “We cannot see well; we have to walk in darkness.” (R9)
- Cataract (*n* = 2; 8%): “When your eye goes white.” (R6)

### Perceived Age Distribution of Ocular Disorders

To assess participants' comprehension of eye disease as a community problem, they were asked which groups they associated with these problems; both populations discussed age. Rural participants said eye disease was a problem of adulthood, with a minority identifying child susceptibility: “Old people don't go to hospital, that's why they have more eye problems.” (R7); “It affects all of us – it does not select just children or adults.” (R10)

On the contrary, every semi-urban interviewee believed eye problems could affect anyone: “Children could have it and elders could not, or vice versa.” (U2)

### Perceptions of Etiology and Transmission of Eye Disease

Participants were asked about transmission, as its interruption is the ultimate goal of trachoma-control programs and local beliefs may either need encouragement or reconditioning to achieve this. Eye disease was widely considered transmissible but underlying theories were diverse. Two rural interviewees (16.7%) identified flies as vectors, which are a recognized mode of transmission in other locations: “Maybe when a fly lands in your eye when you have eye-ache, then it can transport that disease to another person.” (R4)

Three rural participants (25%) also expressed the belief that eye disease originated on other islands, identifying population-to-population transmission: “It used to just exist on Carache, but now it has spread everywhere.” (R5)

Semi-urban participants also identified flies, among other things, as a factor, but all of them concentrated more on hygiene:

If you've worked your hands in something that can cause disease, you cannot put your hands to your eyes without washing them. You must also take care with flies. (U6)

If you don't wash your child's face, it lets *ramela* in the eye and that provokes disease. The mother is responsible for that disease because of a lack of hygiene. (U8)

There were also some less well-informed ideas expressed, including from participants raising correct points, making it difficult to distinguish education from guesswork: “You can get it by looking at one another.” (R1); “It could be eyeliner, wind … the sun.” (U1); “Sometimes, heat can enter your eye.” (U3); “Reading by candlelight.” (U6); “Sometimes we say that it is *La de Poilão* [wool-like tree pollen].” (U9)

Inconsistent ideas regarding transmission were observed at the individual, not community level, perhaps relating to individual experience or education.

### Perceptions of Prevention of Eye Disease

Without necessarily understanding transmission or etiology, it was unanimously believed that eye problems were preventable; one semi-urban interviewee said: “I think eye disease is something more difficult to combat. It is better to prevent before you have it.” (U4)

Semi-urban preventative measures reflected their varied beliefs surrounding transmission. Hand-washing and personal hygiene were unanimously identified as important, although less conventional methods were also described by individuals: “Always use water with soap to wash your face.” (U4); “Avoid reading under candlelight.” (U6); “Maybe if you stay indoors when it's windy, you can avoid it.” (U9)

Conversely, only three rural participants (25%) believed good hygiene was beneficial and they struggled to name any preventative measures, eventually giving basic suggestions: “Avoid shaking hands.” (R1); “Go directly to hospital that's the best prevention.” (R11).

### Perceptions of Effective Treatment of Eye Disease

Understanding local health practices can help gauge confidence in Western-style medicine, an indicator for compliance with mass drug administration, and for the best treatment of eye disease.

As children are the main reservoir of infection, semi-urban participants were asked how they would treat a child with symptomatic eye disease; the overwhelming preference was a western-style clinic (92.3%), expressing distrust of the traditional remedies they were aware of: “You should go to hospital to consult about a child or yourself, I am afraid of using traditional medicine because it is too strong… I don't believe in traditional medicines.” (U13).

Rural participants similarly had faith in hospital medicine, although identified cost as a barrier to healthcare. They reported traditional remedies with more frequency than their semi-urban counterparts (91.7% compared to 30.7%), although they were careful to say that these were used by other people, while they sought hospital treatment and (similarly to the semi-urban interviewees) many rural participants, having identified traditional cures, condemned them:

If someone has eye-ache, you tell them to go to hospital but sometimes they say that they don't have enough money and that's why they can't go. (R7)

All of us have seen people doing it [traditional remedies] but we know it is bad and we ask people to stop doing it. (R11)

Traditional remedies which were reported included washing the face in urine; washing the face with a paste made by grinding the leaves of a local species of tree, or else other flowers, roots or leaves; dripping sap from certain sticks into people's eyes; bathing the eyes in lemon juice or grinding chilli peppers into the eye. Several of these are plainly risky behaviors.

### Beliefs and Perceptions Regarding Oral Medication

Rural participants were asked about their attitudes towards local mass drug administration campaigns, which are periodically conducted using oral tablets. While non-participation is rare, there was mixed understanding of the benefit of the medication: “Taking the pills helps eye-ache and headache a little at the same time.” (R1); “It works when you take pills but are also using ointment in the eyes.” (R2)

### Reported Water Use and Perceptions of Personal Hygiene within the Household

Having placed more importance on personal hygiene in preventing eye disease than their rural counterparts, semi-urban participants were asked about their domestic water use. Several identified water as the most important preoccupation in their daily lives, saying: “Water is life.” (U3); “The first thing I do at home is make sure we have water.” (U13).

Most semi-urban families (69.2%) described water access as easy, using wells or taps. 92.3% kept separated stores, and rudimentary anti-contamination practices were also described:

You cannot use bathing water as drinking water. I have separate bathroom, kitchen and drinking water. (U13)

You can't leave water out as any dirty animal could drink it and then we would catch disease. (U10)

Child-washing behaviors were also investigated and all but one respondent (92.3%) reported washing their children multiple times daily with soap and water: “3 times: the youngest I wash more because they get dirtiest. I put them in a basin and wash them with soap and water.” (U4)

## DISCUSSION

Isolated from mainland Guinea-Bissau, the Bijagos Archipelago has preserved its strong cultural identity and traditional practices, but access to healthcare has been restricted. In this unique setting, disease and infection prevalence and risk factor analyses have been conducted^6^ but sociocultural aspects were previously unexplored.

There has been some informal implementation of community-wide health promotion regarding hygiene sanitation over recent years, alongside community mass drug administration as part of SAFE. Despite this input, participants were often not familiar with the term “trachoma.” Disease terminology is clearly non-essential for disease control, but familiarity with the term “trachoma” may provide a focus for future interventions and help link, and therefore consolidate understanding of, the apparently unrelated appearances of active and chronic pathologies. Awareness of trachoma terminology has been significantly improved in Ethiopian and Malian communities by regularly radio broadcasting trachoma-related public health messages.^18^^,^ 19 These methods of promotion and education could be viable and sustainable on Bubaque, where most families have access to radios^6^ and there are daily community radio programs in the local dialect.

Without necessarily using the term “trachoma,” both interview series described active and chronic trachomatous symptoms and appreciated that a problem existed within their communities. However, they did not seem to associate childhood infection with adult scarring symptoms, a factor which has previously been shown to limit antibiotic uptake in community treatment campaigns.^20^ The acceptance and recognition of trachomatous symptoms as a public health problem is important regarding implementation of trachoma elimination activities. In other settings, where communities lack this recognition, there has been refusal to comply with treatments and interventions.^11^^,^ 21

Similar research in the Gambia^12^ concluded that the lack of connection between childhood active infection and trichiasis-induced adult blindness was a major obstacle to SAFE implementation. It also postulated that traditional remedies persisted due to local perceptions of trachoma's etiology and natural history, despite observing Western medicine effecting a cure. While this research draws interestingly similar conclusions, direct comparison is difficult in the face of this work's small sample size and different setting.

Participants identified healthcare consultations as a potential opportunity for information dissemination, where healthcare staff could provide education alongside treatment. Communication may be a problem however; the majority of formally-trained healthcare professionals are recruited from the capital, Bissau, and speak Kriolu (the national lingua franca) whereas on the islands, particularly in the older generations, Bijogo is the predominant local language. In addition to potential language barriers, healthcare staff retention and continuity of care in health facilities and communities is frequently an issue. In a similar setting, the Gambian National Eye Care Programme (NECP) trained community-elected individuals called Nysteros (Friends of the Eye) and demonstrated they could successfully disseminate good eye health practices.^22^ The Guinea-Bissau national trachoma control program similarly plans to recruit and train healthcare staff from local Bijagos communities, the results of which would bear further investigation.

Semi-urban populations thought eye disease could affect all age groups, children being particularly high risk. Rural populations associated eye problems with the elderly, perhaps reasonably, given that end-stage trachoma is more externally apparent than the often asymptomatic active infection. None of the participants, however, appreciated the link between childhood infection and the scarring sequelae of adulthood; this lack of association may impede compliance with mass antibiotic administrations, delay treatment-seeking behavior and hinder uptake of disease control behaviors.^14^^,^ 23 Future public health education might focus on raising awareness of eye disease in children, the concept of asymptomatic disease and explaining the natural history of trachoma to nurture understanding.

Both populations provided an interesting array of trachoma etiologies, most participants having both accurate and inaccurate ideas. The responses provide useful insight; explanations were logical and reasonable, often based on observation and experience. The lack of consensus among participants suggests there is no prevailing theory regarding etiology and with no evidence of a firmly-held underlying belief system which might espouse unhealthy practices, it is reasonable to assume that future education campaigns will not face significant resistance if they conform to local logical thinking.

While rural populations agreed that eye disease was preventable, no potential measures were suggested. Semi-urban populations, however, unanimously believed that face washing and good personal hygiene, crucial to the SAFE strategy, could prevent eye problems; even associating child cleanliness with good parenting. They also demonstrated good knowledge of safe water usage, keeping separate stores and taking precautions to avoid contamination. The uniformity of these opinions implies previous hygiene promotion may have taken place in semi-urban communities. Social-acceptability bias may have led to over-reporting of favorable hygiene behaviors^11^ however, as children routinely had dirty hands and faces with nasal and ocular secretions, which are implicated in trachoma. Stressing the importance of these behaviors in preventing trachoma might encourage the translation of reported child-washing into hygienic practice, in turn reducing transmission;^8^ washing at least once daily has been shown to significantly reduce the odds of infection (odds ratio 0.76, 95% CI 0.57–0.96).^24^ The use of towels and soap are similarly significant, however local access to these is limited and provision would be less sustainable. Tailoring public health campaigns to the semi-urban and rural populations' existing knowledge levels could also be useful in optimally improving understanding and uptake of disease prevention interventions.

Both interview series reported faith in “Western” medicine, however islanders' practices such as attempting to put oral tablets into the eyes raises the issue of the need for comprehension in the presence of compliance. Reassurance of the efficacy of systemic treatment, even in apparently healthy people, may be an important tool. With regard to treatment-seeking behavior, semi-urban communities unanimously reported going to the nearby hospital first. Rural interviewees, who live much further away, tended to seek traditional remedies, only attending hospital if this failed. This difference probably reflects the more traditional rural lifestyle, their distance from the hospital and financial barriers. Currency has little role in the subsistence economy of rural villages and villagers are therefore deterred from seeking hospital care by the prospect of unaffordable fees. These factors make the addition of trained village volunteers, like the Nysteros, into communities an even more pertinent recommendation.

Rural participants acknowledged widespread use of traditional remedies despite recognizing their potential harm and rarely admitting personal use. Reasons for their persistence may include their role in rural culture, the capacity for non-monetary payment and their ready availability.^25^ Further anthropological investigation into this area and decision-making when seeking treatment is planned; complex health beliefs influence this behavior and provision of modern eye services does not guarantee their use.^13^^,^ 26 Education about the risks of traditional treatments may reduce their use.

### Limitations

Several limitations are recognized in this research. The sample size was small, with a total of 25 participants (12 purposively sampled and with some communities unrepresented). This is unlikely to be fully representative of the range of beliefs held by the community. The sample size is effectively further reduced by comparing one interview series with the other. Nonetheless, discernible patterns emerged, indicating trends in local beliefs. These findings could be validated with further interviews.

Being unmatched also limits the series comparability, but having proven viability with the first series, the second focused on childcare providers as their opinions could shape interventions aimed at interrupting transmission among children. Despite participant populations differing systematically, they are still representative of their communities to a degree appropriate for basic comparison. Furthermore, although each series had some series-unique questions, this reflects the pilot nature of the first series and the flexibility of semi-structured interviews. While precluding population comparison, it did explore opinion within each population.

An unavoidable language barrier resulted in interviews being conducted by locally recruited and Kriolu-fluent field assistants. Despite adequate training and briefing, familiarity and experience in the use of qualitative methods takes time to acquire. The impact of this inexperience was minimized by researchers where possible, and proficiency developed. Themes may also have been lost or corrupted due to indirect translation. Ideally, fully trained qualitative researchers fluent in the local dialect should conduct interviews of this kind, but this was beyond the scope and resources of the current study, whose primary aim was to undertake a pragmatic investigation to gain insight into these communities' perceptions of trachoma that may be relevant to the successful implementation of SAFE.

Researcher association with western healthcare may also have introduced social acceptability bias. Future interviews might be conducted by local researchers to address this, but researcher bias is a common and largely unavoidable limitation in qualitative research.

### Conclusions and Recommendations

This is the first study to examine sociocultural beliefs relating to trachoma in these populations, and the results are likely to have implications for future SAFE implementation. Basic public health knowledge was present and eye disease, described in participants' own terms, was a recognized community concern. There were varied beliefs surrounding transmission and prevention; some were in line with WHO policy, while others, though logical, differed markedly. Additionally, faith in western healthcare was reportedly greater than in traditional remedies, which may be important for education programs and mass drug administration. Semi-urban participants particularly described good hygiene practices, and encouraging these in both rural and semi-urban populations may be important in the elimination of blinding trachoma. There is a paucity of data demonstrating the impact of F and E interventions on the reduction of transmission of infection or prevalence of trachoma. Rigorous studies assessing the impact of F and E interventions after implementation are required.

Potential differences between rural and semi-urban populations indicate that SAFE implementation and education could be tailored to different populations. Such differences merit further investigation. Investigating the origins of health beliefs, perpetuating factors for traditional medicine, perceived barriers to healthcare and anthropological study may all enlighten our understanding of this population's beliefs further, and observational studies could reveal participants' actual behavior. Such results could add to this research's findings and further refine local SAFE implementation to help eliminate blinding trachoma on the Bijagos.

## Figures and Tables

**FIGURE 1A.**
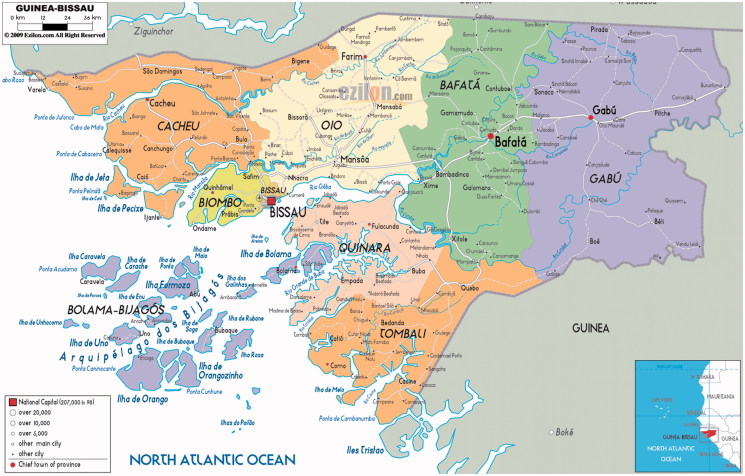
Map of Guinea-Bissau, showing the Bijagos Archipelago. Source: Ezilon Maps, 2014.

## APPENDIX 2

### Semi-Structured Interview Topic Guide (Series 1, Rural Communities)

Researcher: Katie Thompson

Field Assistant: Benedito Icossoboc

Translator: Duarte (English professor in local school)

March 2012: Bubaque island, Bijagos Archipelago, Guinea-Bissau

1. Knowledge of trachoma  *If the term trachoma is not recognized, explore knowledge and descriptions of known eye disease. If nothing offered by participant, describe and show pictures of trachomatous disease*.
2. Disease transmission
3. Disease prevention strategies
4. Treatment:
  - What do they do when they/their families have eye problems
  - What treatments are they aware of other people using
  - Perceptions towards western treatments and hospital care
5. Beliefs about blindness and its causes

## APPENDIX 3

### Semi-Structured Interview Topic Guide (Series 2, Semi-Urban Community)

Researcher: Harry Hutchins

Field Assistant: Aramato Baio

Translator: Duarte (English professor in local school)

March 2013: Bubaque island, Bijagos Archipelago, Guinea-Bissau

1. Introduction
  - Introduce self and field assistant
  - Confirm consent
  - Briefly reiterate purposes of interview and general subject matter
2. Introduction to washing behavior
  - How many children live here?
  - Who is responsible for washing them?
  - How often do you wash the children?
  - How do you wash their faces? What do you use to do so?
3. Water
  - Is it straightforward for you to obtain water?
  - What do you use the water for?
  - Do you have water in your house specifically for washing yourselves?
  - Do you always have some?
  - Why do you/do you not?
4. Attitudes to child cleanliness
  - Are there any problems you know about associated with children having dirty hands or faces?
  - How do you think dirty hands/faces cause them?
  - Do you know about any effects it has on health? [If not mentioned]
  - Do you know about any effects it has on eyes? [If not mentioned]
5. Eye health
  - What do you think causes eye problems? How?
  - Do you think there is any difference with children?
  - Do you know of any ways to prevent these problems?
  - If your child had an eye problem, what would you do? What do other people do? Why?
  - Do you know anything about trachoma?
6. Other
  - Relay perceived major points back to participant to confirm
  - Thank participant

*Note*: The question on oral medication was asked only to rural participants and the question on domestic water use only to semi-urban participants. The former was removed from the subsequent rural interviews as the very high rate of compliance with oral medication made the authors question the direct usefulness of this information. The water usage question was added on the basis of subsequent quantitative study suggesting that water use was associated with the presence of trachoma^6^ and specific hygiene practices (Thompson K, et al. Unpublished data).
